# Esophageal compression by a common left pulmonary venous trunk

**DOI:** 10.1259/bjrcr.20200007

**Published:** 2020-06-23

**Authors:** Daniel Z Mogel, Donald P Kotler, Mark Guelfguat

**Affiliations:** 1 New York Institute of Technology College of Osteopathic Medicine, 101 Northern Boulevard, Glen Head, NY 11545, United States; 2 Department of Medicine, Jacobi Medical Center, Division of Gastroenterology, Albert Einstein College of Medicine, 1400 Pelham Parkway South, Bronx, NY 10461, United States; 3 Department of Radiology, Jacobi Medical Center, Albert Einstein College of Medicine, 1400 Pelham Parkway South, Bronx, NY 10461, United States

## Abstract

Dysphagia is a symptom with diverse etiologies including luminal narrowing of the esophagus and motility disorders. Arterial vessels are known to compress the esophagus and cause luminal narrowing. However, identifying a pulmonary venous compression of the esophagus rarely occurs in a patient with dysphagia. The technology available at the time of the few prior case reports published more than three decades ago limited the analysis of the pulmonary vessels. We report a case that utilized CT-angiography as well as multiplanar reconstructions and three-dimensional imaging to demonstrate that esophageal compression in the patient presenting with dysphagia was caused by a large left common pulmonary vein.

## Introduction

Dysphagia is a symptom that can be caused by various entities, including obstructing masses as well as motility disorders. Our case detected what was initially suspected to be a submucosal esophageal mass on barium esophagram. Esophageal lumen compression may be caused by masses, such leiomyomas or duplication cysts, as well as vascular abnormalities.^1^ A vast majority of the vascular causes are arterial in nature. Rarely, pulmonary venous compression of the esophagus has been demonstrated on imaging studies utilizing angiocardiography.^2–4^ One prior study utilized CT-angiography and found that an enlarged left inferior pulmonary vein was causing esophageal compression in an asymptomatic patient.^5^ We report a case of a patient presenting with dysphagia that utilized CT-angiography to demonstrate esophageal compression caused by an enlarged left common pulmonary vein.

## Case report

A 70-year-old female with history of hypertension presented to the emergency department with complaints of intermittent difficulty swallowing liquids, especially when ingesting a large bolus. She had no further relevant past medical history and denied smoking and alcohol use. Her symptoms had started 1 year prior to presentation and occurred once a month. She endorsed history of intermittent dysphagia symptoms while attempting to swallow liquids and rice, as well as a mild loss of appetite. She denied odynophagia, globus sensation, weight loss, abdominal pain, hematochezia, and dyspepsia. Physical exam was unremarkable. Initial clinical differential diagnosis of intermittent dysphagia with solids/liquids would seem most consistent with a motility disorder. Neck radiographs were ordered to rule out foreign body. Double contrast barium esophography, as well as endoscopy, were ordered to evaluate for a possible motility disorder or esophageal compression that could be causing the dysphagia.

**Figure 1.**
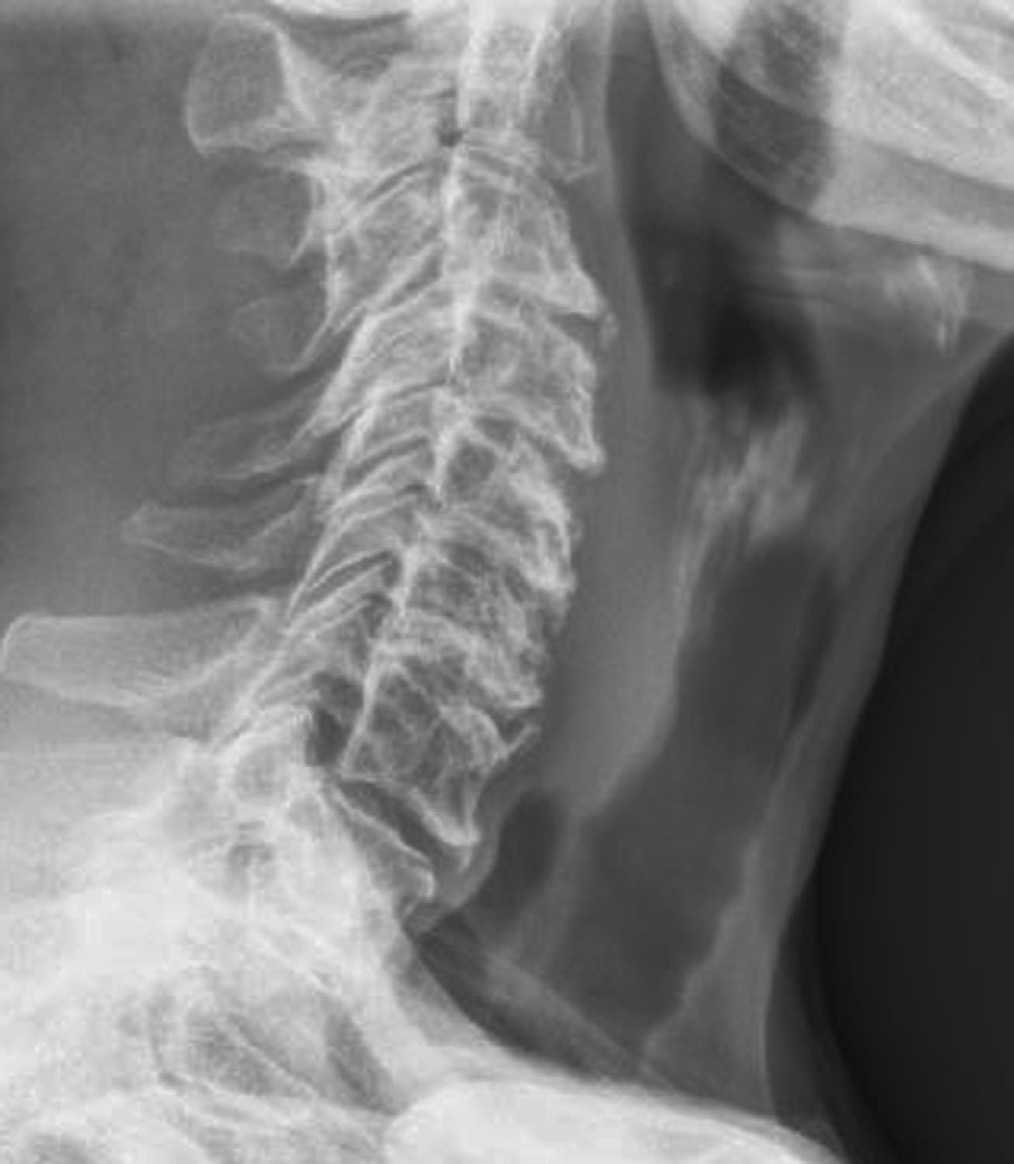
Lateral neck radiograph reveals multilevel mild anterior cervical spine osteophytes. They do not produce mass effect on the esophagus.

**Figure 2.**
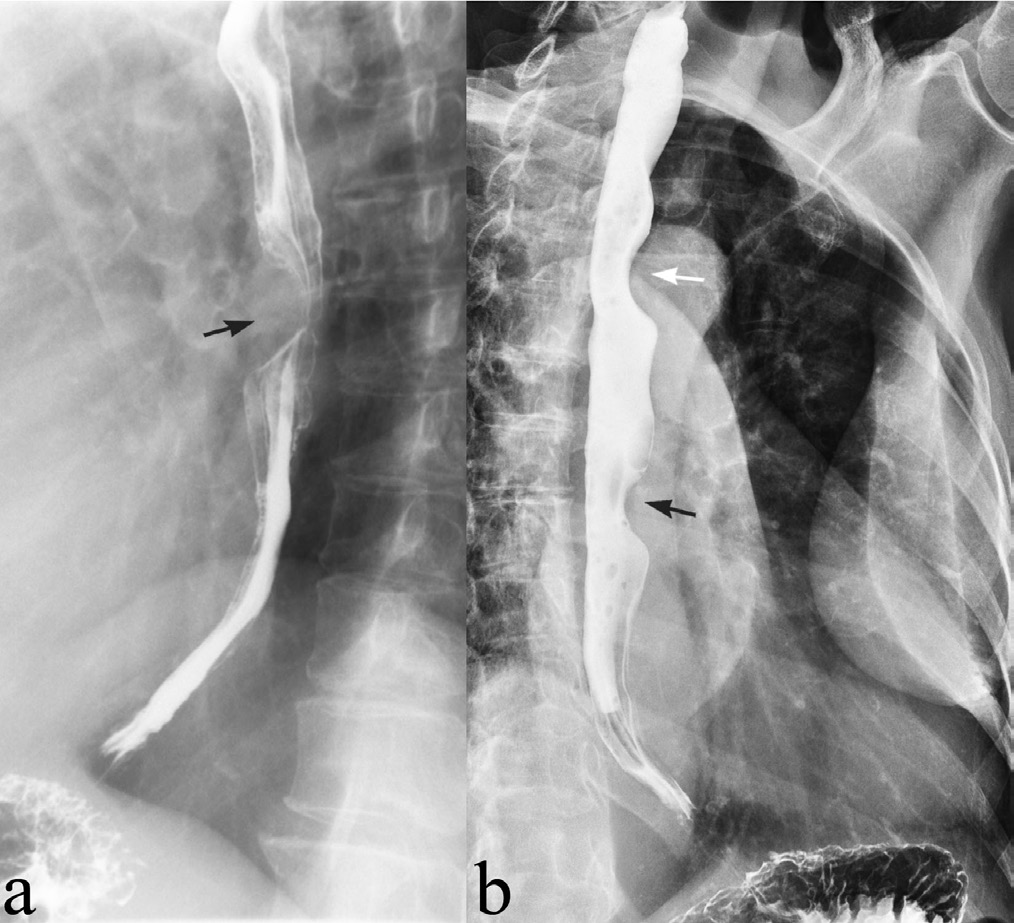
(a) Double contrast barium esophagography showing smooth anterior compression on the lower esophageal lumen (black arrow). (b) An overhead radiograph during the study reveals that the lower esophageal compression remains during barium ingestion (black arrow). The upper esophageal compression (white arrow) is due to aortic arch.

Neck radiographs showed multilevel moderate anterior cervical spine osteophytes without displacement of the anterior prevertebral soft tissues. No radiopaque foreign body was seen (Figure 1). Double contrast barium esophagography revealed anterior compression on the lower esophageal lumen with smooth margins and well-defined angles that remained during barium ingestion (Figure 2). No strictures or mucosal defects were seen elsewhere. This appearance was suggestive of submucosal mass extending into the lumen. Additionally, decreased primary peristalsis was noted within the esophagus.

**Figure 3.**
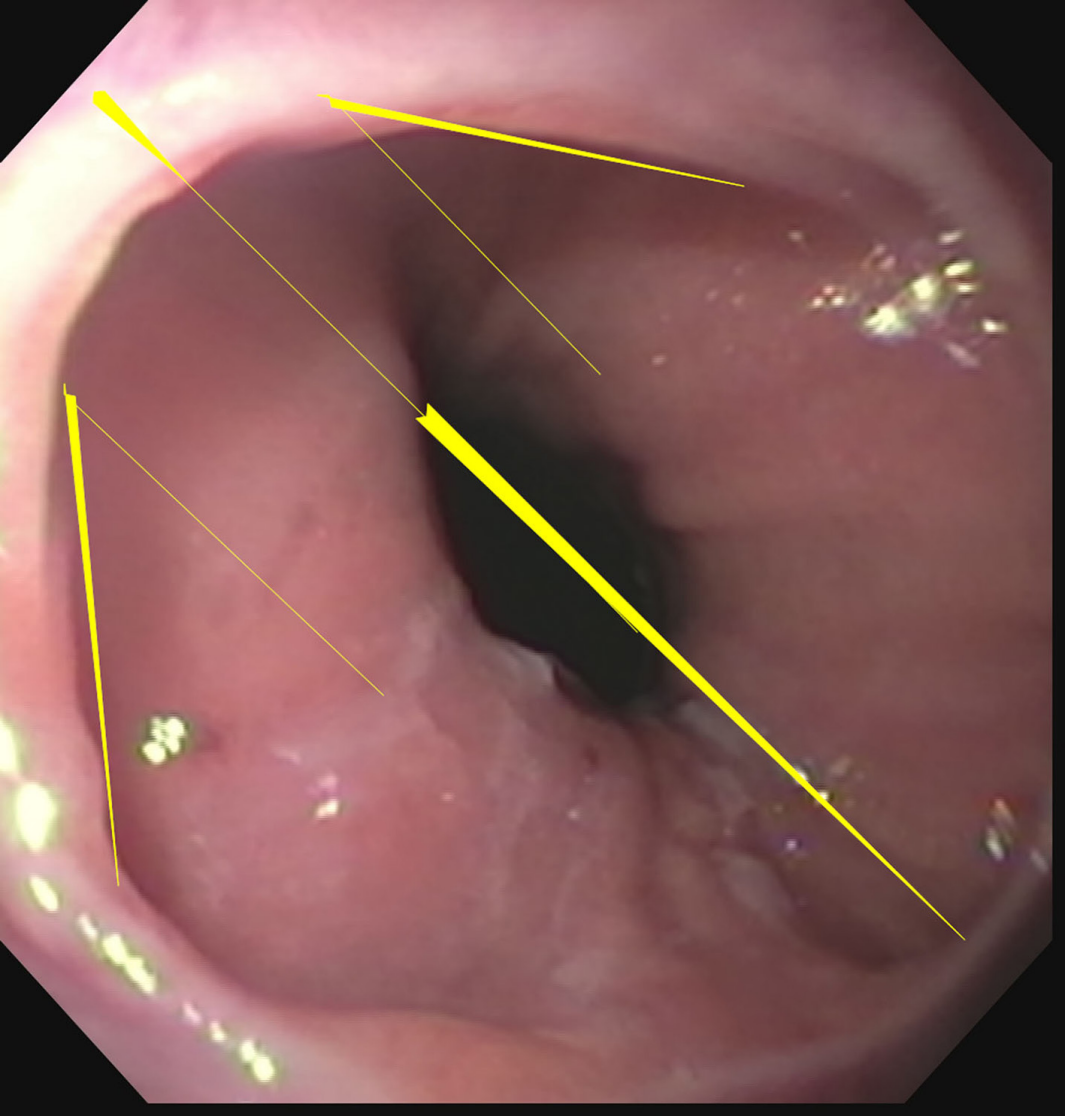
Endoscopic image demonstrates the perimeter of the Schatzki ring outlined by the thin short yellow lines. The longitudinal diameter of the esophagus is indicated by the long thick yellow line.

Endoscopy revealed a non-obstructive Schatzki ring 2 cm proximal to the gastroesophageal junction. The exact measurements of the distal esophageal diameter were not obtained during endoscopy. However, since the endoscope passed into the stomach without difficulty, this indicates that the luminal size was greater than 12 mm, which is the diameter of the endoscope. The Schatzki ring seen endoscopically (Figure 3) demonstrated minimal narrowing of the esophageal lumen and was not apparent on the esophagography. A chest CT scan was ordered for further evaluation of the esophageal compression.

**Figure 4.**
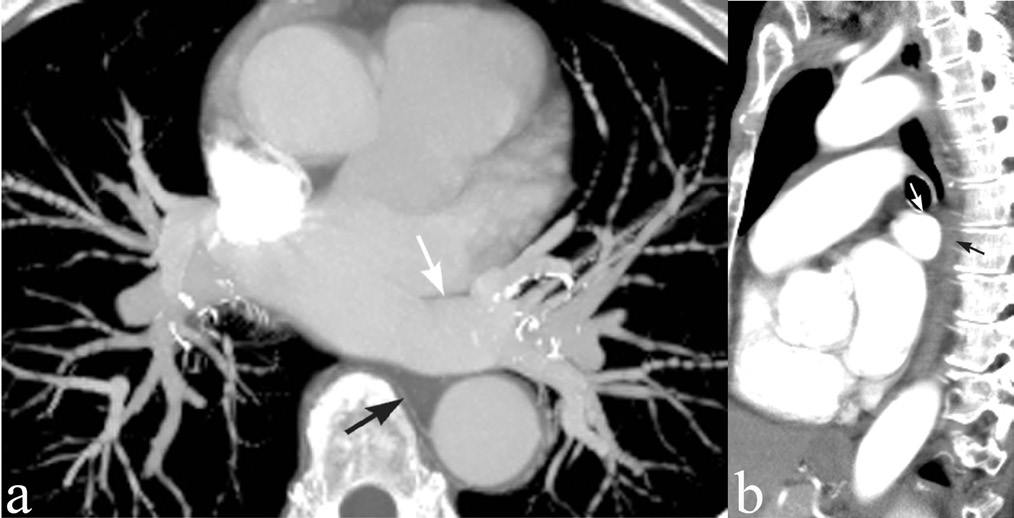
(a) Axial CT with intravenous contrast, thick MIP. A common venous trunk is draining the left pulmonary veins (white arrow). It passes ventral to the thoracic esophagus (black arrow). At that location, the esophagus is wedged between the common venous trunk, aorta and vertebral body. (b) Sagittal MPR verifies the relationship of the left common venous trunk (white arrow) and esophagus (black arrow) seen on the prior CT image and confirms that the venous structure produced the compression seen on the fluoroscopic examination. MIP, maximum intensity projection; MPR, multiplanar reconstruction.

**Figure 5.**
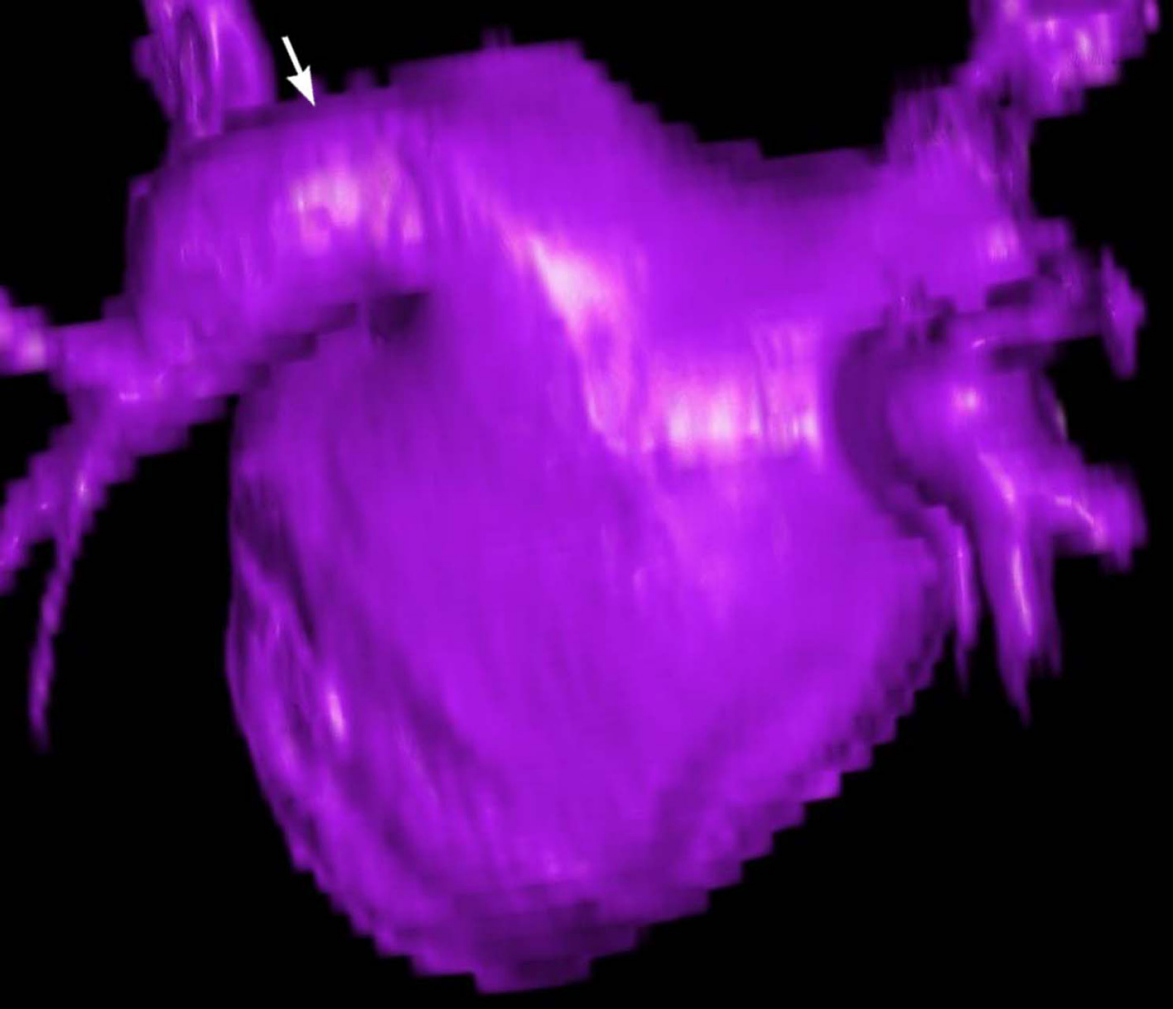
A 3D VR image of the left atrium viewed from the back of the heart. The horizontal course of the common left venous trunk is apparent (white arrow). 3D VR, three-dimensional volume rendered.

Axial CT with intravenous contrast demonstrated a common venous trunk draining into the left atrium. The single trunk is passing horizontally, ventral to the lower thoracic esophagus, at the level of the anterior esophageal compression detected with barium esophagography. Sagittal multiplanar reconstruction (MPR) confirmed that the venous structure was the culprit producing the compression seen on the fluoroscopic examination (Figure 4). This 1.8 cm long common venous trunk was formed by the confluence of the left lower and upper lobe veins (Figure 5). Diameter of this venous channel is greater than that of normal individual pulmonary veins. No esophageal or mediastinal masses were seen.

Based on this work-up, the patient was determined to have an obstruction in the lower esophagus. Due to lack of significant morbidity and intermittency of the symptoms, the patient was advised to continue follow-up as an outpatient rather than consider surgical management. During a follow-up appointment with a gastroenterologist 7 months after the initial presentation, the patient reported having stability to mild improvement of dysphagia with most foods except for rice. No interval weight change was detected. She was suggested to continue following with her primary care physician.

Consent to publish this case report from the patient could not be obtained. The patient was uncontactable despite exhaustive attempts being made. The case has been entirely de-identified.

## Discussion

This patient’s esophageal compression was originally thought to be caused by a submucosal mass based on smooth margins and defined angles on the esophagram. Common etiology of compressive submucosal lesions includes esophageal duplication cysts, leiomyomas, and gastrointestinal stromal tumors.^1^ Additionally, compression of the esophagus can be associated with aberrant vascular structures, which has been well described in medical literature. These are generally seen in children, and are caused by anomalous arterial vessels, such as double aortic arch.^6^ Such conditions often present with dysphagia and may persist into adulthood, such as so-called dysphagia lusoria, seen in a setting of aberrant origin of the right subclavian artery from the aortic arch.^7^

Dysphagia in a setting of pulmonary venous compression of the esophagus has been seldom reported. Esophageal compression by left pulmonary venous structures in a setting of dysphagia has been previously identified angiographically. These veins were described as left superior pulmonary vein, left inferior pulmonary vein and a common left pulmonary vein.^2–4^ A report containing CT imaging of this entity describes the vessel as the left inferior pulmonary vein. However, this was an incidental finding and was not associated with dysphagia.^5^ To the best of our knowledge, there has been no report using CT data identifying a common pulmonary trunk compressing the esophagus with the patient exhibiting dysphagia.

Common left pulmonary venous trunks are a normal variant. This variant pattern has been found to be present in a range of 14–34% on retrospective analysis of routine CT studies.^8,9^ Our case confirms that mass effect on the esophagus was by the common venous trunk. The contribution of this case report is to demonstrate that a left common venous trunk can undoubtedly cause esophageal compression. This is illustrated clearly via use of MPRs and three-dimensional imaging post-processing techniques which have been developed owing to advances in CT technology since the prior publications.

It may be difficult to establish causality of dysphagia, as symptoms are subjective and often intermittent. Included in the differential diagnosis for intermittent dysphagia are anatomic causes of esophageal compression as well as motility issues.^10^ Intermittency of dysphagia in our patient could indicate involvement of an underlying unspecified motility disorder. Since the radiologic studies in our patient identified both compression of the esophagus as well as decreased primary peristalsis, it is possible that the clinical picture is due to combination of dysmotility and observed vascular esophageal compression. We believe that the effects of the esophageal compression may be intermittently exacerbated by our patient’s non-specified motility disorder. Unfortunately, evaluation of the cardiac function, particularly pulmonary venous pressures, was not obtained in our patient. Therefore, effect of cardiac function on dysphagia cannot be demonstrated in this case.

In conclusion, we report a patient with dysphagia who was found to have esophageal compression by a common left pulmonary vein. To the best of our knowledge, CT appearance of this entity has not yet been reported.

## Learning points

1. There are few pulmonary venous system variations. One of the most frequently seen is the common left venous trunk draining the superior and inferior veins.
2. Esophageal compression by aberrant arterial vasculature is known cause of dysphagia. It is also important to consider esophageal compression by a venous anomaly or variant.
3. CT with MPRs and three-dimensional imaging is a helpful tool in evaluation of the causes of an extrinsic esophageal luminal compression, especially by vascular etiologies.
